# Differences in antibiotic use and knowledge between adolescent and adult mothers in Ecuador

**DOI:** 10.12688/f1000research.2-108.v2

**Published:** 2013-07-08

**Authors:** Arturo Quizhpe P, Martyna Gassowski, Lorena Encalada T, Francoise Barten

**Affiliations:** 1 International Center for Health Systems Research and Education, Radboud University Nijmegen Medical Centre, 500 HB, Nijmegen, Netherlands; 2 Faculty of Medical Sciences, University of Cuenca, Cuenca, EC010107, Ecuador; 3 Radboud University Nijmegen Medical Centre, 6500 HB, Nijmegen, Netherlands; 4 Department of Internal Medicine, University of Cuenca, Cuenca, EC010107, Ecuador; 5 Department of Primary and Community Healthcare, Radbound University Nijmegen Medical Centre, 6500 HB, Nijmegen, Netherlands

**Keywords:** Child care, child health, maternal educational status, patient education, antibiotics, Latin America

## Abstract

**Objectives:** To investigate the differences in antibiotic use and knowledge between adolescent and adult mothers of children under the age of 5 years in Ecuador.

**Methods:** A cross sectional study was performed in four health centers and hospitals. Mothers of children under five years, seeking medical attention their child's upper respiratory tract infection (URI), were included. The data was collected through interviews, using a structured questionnaire. The questionnaire covered the topics knowledge of antibiotic treatment, risk and resistance.

**Results:** 777 mothers were included in the study, of which 15.8% were adolescent and 84.1% adult mothers. There were significant differences in the social and economic characteristics of the mothers (p ≤ 0.05), with adolescent mothers being more likely to have an incomplete high school education and lack of basic services in their home. Significant differences between these groups were found in adherence to treatment, knowledge about risks associated with antibiotic use, and having heard of antibiotic resistance. Among the adult mothers, 83.5% reported correct adherence, 28.5% were knowledgeable about risks associated with antibiotic use, and 29.3% had heard of antibiotic resistance. Among the adolescent mothers, these numbers were 75.4%, 15.0%, and 19.8%, respectively.

**Conclusions:** To develop successful interventions, it is crucial to understand the factors causing differences in antibiotic use and knowledge between mothers.

## Introduction

Antibiotics are potent drugs, which since their discovery have saved millions of lives. However, when used incorrectly, they can pose a health risk to the patient, as well as contribute to the development of bacterial resistance to these drugs. Considering the already alarming levels of resistance reported worldwide ^1, 2^ an improvement in use, in terms of indications for treatment, choice of antibiotic, dosage, duration and enhanced knowledge, are crucial steps towards containment of the problem of resistance.

The majority of antibiotics are consumed in the community ^3^, and in this setting there is plenty of opportunity for incorrect use. Particularly in countries with lack of access to and availability of health care, poor drug control, and lack of education of the population, self-medication is often common practice ^4, 5^. The high cost of medicines, incomplete treatment courses, and patient pressure on the physician for a prescription, contribute to the misuse of antibiotics. Studies from both developed and developing countries show that misconceptions about antibiotics and their use are prevalent in the lay populations ^6– 9^. It is for example frequently thought that antibiotic treatment is indicated in conditions such as the common cold, which in most cases is a condition of viral etiology ^4– 6^. The financial aspect also influences the use of antibiotics, and poor patients will often not be able to afford a doctor visit or full treatment ^10^. On the other hand, many health care systems do not make an effort to educate either the lay population about the importance of correct use of antibiotic, or the physicians about how to best deal with these problems.

In Ecuador, little is known about the use of antibiotics and the relevance of educational efforts made. In a review of the use and misuse of antibiotics in Latin America, M. J. Wolff described an "antibiotic culture" where treatment often is thought to be indicated at any sign of a suspected infection, which results in frequent self-medication ^4^. In order to be able to address this and other incorrect antibiotic use through interventions, it is important to first identify the causes of these behaviors in the local context, as well as the differences between different groups in the society. Preschool children are a group that are highly exposed to antibiotics in the community ^11, 12^. However, in the case of such young children, the final decision about antibiotic treatment is taken by the child’s care giver, usually the mother. In Ecuador, despite an over-all decreasing fertility rate, the group of adolescent mothers is steadily increasing ^13^. Thus, the aim of this study was to investigate whether there are differences in antibiotic use and knowledge between adolescent and adult mothers, seeking care for an upper respiratory tract infection (URI) in their child, aged less than five years. URIs are the leading cause of morbidity and doctor consultations in children under the age of five in Ecuador ^14^, and it was therefore chosen as a reference condition for the study.

## Methods

### Ethics

Patients were informed about the study and its goals and oral informed consent was obtained.

### Study sites

This cross sectional study was performed between February and April 2011, in four health centers and basic hospitals in the provinces of Azuay and Guayas in southern Ecuador. The sites were selected to represent a variety of ethnicities, local cultures, and climatic regions. The selected study sites are located in the highlands and in the coast and cover both urban and rural populations. Table 1 gives an overview of the characteristics of each of the health facilities and the populations they cover.

**Table 1.** Basic characteristics of the population covered by each health facility.

Study site	Geographical location	Population covered	Urban Population (%)	Main economical activity (%)
Basic hospital of Naranjal	South west of the Guayas province	53.800	67	Agriculture (49)
Basic hospital of Sígsig	South east of the Azuay province	24.635	14	Agriculture (56)
Health center ”el Paraiso” (city of Cuenca)	North of the Azuay province	26.672	100	Private sector employment (80)
Health center of Nábon	South of the Azuay province	18.053	58	Agriculture (58)

### Study design and sampling

The data analyzed in this article came from a larger cross-sectional survey aimed at investigating perceptions of disease severity, treatment and antibiotic use in caregivers of children under the age of five. The data was collected through interviews, using a structured questionnaire. All mothers of children under five years of age, seeking medical attention in one of the study sites for a respiratory tract infection in the child, were eligible for the study. Due to low numbers of attendance in the health center in Nabon, the approach was changed and the participants from this study site were recruited during medical visits in the home. The aim was to include 200 participants from each of the study sites.

Study questionnaire (Spanish version) Questionnaire investigating perceptions of disease severity, treatment and antibiotic use by adolescent and adult mothers of children under the age of five.

### Outcome measures

The questionnaire included questions about demographic and socioeconomic characteristics of the child, mother and family, and questions about antibiotic knowledge and use by the mother. The main outcome measures were if the mother had ever used antibiotics without previously consulting a physician; always gave the complete treatment course to her child; knew of any risk associated with antibiotic use; and had ever heard of antibiotic resistance. The secondary outcome measures were the source of antibiotics if not consulting a physician before use; reason for giving incomplete treatment course if ever doing so; source of information about risks associated with antibiotic use if having this knowledge; and source of information about antibiotic resistance if they had this knowledge.

### Statistical analysis

The statistical analysis preformed consisted of a descriptive analysis of the study population. To assess the significance of the differences between the adolescent and adult mothers, a Chi-square test was used, with level of significance set to p≤0.05. The data were analyzed using SPSS 17 for Windows.

## Results

In total, 777 participants were included in this study. Of these, 123 (15.8%) were adolescent mothers (aged 19 years or younger at the birth of their child), while 654 (84.2%) were adult mothers. The mean age of the adolescent mothers was 17.5 years (s = 1.3), and 27.1 years (s = 5.3) for the adult mothers at the time of the study. Table 2 shows the basic characteristics of the mothers and of their living conditions.

**Table 2.** Basic social and economical characteristics of the mothers and of their living conditions.

	Adolescent mothers		Adult mothers		
	Number	%	Number	%	P-value
Completed high school education or higher	29	23.6	274	41.9	0.001
Live in rural area	74	60.2	692	45.3	0.003
Access to basic services ^a^	31	25.2	287	43.9	0.000
More than two persons/bedroom	39	31.7	245	37.5	0.224
Two or more children <5 years of age in the family	34	27.6	275	42.2	0.003
Total family income of <263 USD per month ^b^	69	56.1	298	45.8	0.036

^a^ Basic services is defined as having electricity, running water and connection to a sewage system in the home.

^b^ Gross minimum wage in Ecuador.

The groups differed significantly in social and economic characteristics. Adolescent mothers were more likely to have an incomplete high school or primary school education and to live in a rural area. They also were more likely than adult mothers to live in a home lacking access to at least one of the basic services (electricity, running water or a connection to a sewage system). In addition, the number of children was lower in the group of adolescent mothers.

Adult mothers were significantly more likely to have at least two children under the age of five in the family and they were also more likely to have a family income exceeding one minimum wage per month, than were the adolescent mothers.

Table 3 shows the responses to the questions concerning antibiotic use and knowledge of the mothers. For all questions, less than 1% of the data was missing. Having used antibiotics without previously consulting a physician was common in both groups, with 30 (24.6%) of the adolescent mothers and 191 (29.2%) of the adult mothers having ever done so, but no significant difference between the groups was found. However, a significant difference was found in adherence to treatment, where 543 (83.5%) of the adult mothers stated that they always gave the complete antibiotic course to their child, compared to 92 (75.4%) of the adolescent mothers. The reasons behind not completing a treatment were also investigated, and the most frequently given reason in both groups was that the child was feeling better, stated by 19 (61.3%) in adolescent mothers and 60 (55.0%) in adult mothers. However, no significant differences were found between the groups in this aspect (p< 0.061).

**Table 3.** Antibiotic use and knowledge of the mothers.

	Adolescent mothers		Adult mothers		
	Number	%	Number	%	P-value
Has ever used antibiotics without previously consulting a physician	30	24.6	191	29.2	0.295
Always gives complete treatment course to the child	92	75.4	543	83.5	0.031
Knows of any risk associated with antibiotic use	18	15.0	185	28.5	0.002
Has heard of antibiotic resistance	24	19.8	191	29.3	0.032

Of the adult mothers, 185 (28.5%) stated that they knew of any of the risks associated with antibiotic use, such as antibiotic resistance, allergy and secondary effects compared to 18 (15.0%) of the adolescent mothers, a difference that was significant.

24 (19.8%) of the adolescent mothers and 191 (29.3%) of the adult mothers had heard of antibiotic resistance, and this difference was significant. The majority in both groups (56% of the adolescent mothers and 68.1% of the adult mothers) stated the physician as the source of this knowledge. There was no significant difference found between the groups either for this source, or for others.

Perceptions about the use and knowledge of antibiotics in adolescent and adult mothers in Ecuador The data presented here was collected through interviewing mothers using a structured questionnaire seeking medical attention for their child (<5 years old) with upper respiratory tract infection in four health centers and hospitals in Ecuador. Adolescent mothers more frequently misused and knew less about antibiotics compared to adult mothers, though knowledge values were low in both groups. Data in the following columns were not included in the analyses in this article: C, G, K, P-T, AJ-CK, CO, DH-DY.

## Discussion

This study found that adolescent mothers more frequently used antibiotics incorrectly and had less antibiotic knowledge compared to adult mothers. A number of differences in the characteristics of the mothers and their living conditions were identified, showing a tendency of the adolescent mothers to have a lower level of education and income, and to be more likely to live in rural areas.

In regard to the level of education of the mothers, as one of the determinants of this study demonstrates, only 23.6% of adolescent mothers completed high school compared with 41.9% of adult mothers. A study in Cuba on educational level of mothers and their knowledge, attitudes and practices concerning acute respiratory infections in their children concluded that the most important variable for the adequacy of knowledge was the educational level, which highlights the fundamental and positive influence of this factor on the preventive and curative care that mothers provide for their children, results by regression of age of the mother and level of education were RP 0.91 95% (0.85–0.98) ^15^.

Another study in Spain on appropriate use of antibiotics in primary health care points out that an adequate level of beliefs in parents about the indications of antibiotic use in several illnesses are associated with a high level of parental education (Odds Ratio, 2.04; 95% CI, 1.16 -3,06). In relation to the level of education, 45.8% of mothers had only a primary school education. 73 children (21%) received antibiotics in the 3 months before the study. 27 parents (7.8%) bought antibiotics without prescription. 82 respondents (23.6%) stated that their pediatrician did not prescribe antibiotics in situations where they expected them to do so ^16^.

As found in our study, the education of mothers is an influential factor in the knowledge and use of antibiotics in case of URIs, because it allows greater access to the basic information on health education in order to recognize the symptoms and signs that require urgent attention at a health care centre and adopt healthier lifestyles to decrease the level of child morbidity. Another study in 2008–2009 in the Ahmedabad district in India, found that the prevalence of acute respiratory tract infections (ARI) was higher in low social classes (III, IV and V) (26.56%), illiterate mothers (24.4%) and first-time mothers (23.9%) and those living in overcrowded houses (28.5%). At the same time, the prevalence of ARI was lower in urban areas (17.2%) as compared to rural areas (26.8%). This difference is more likely due to a lack of availability of basic health services and other associated factors like overcrowding, low socio-economic status, absence of cross ventilation and indoor air pollution.

About one third (30%) of children belonged to upper social classes (I, II) and the remaining were in low social classes (III, IV, V). According to social class, prevalence of ARI was higher in lower social classes (III-31.4%, IV-22.1%, and V-26.2% respectively) and was statistically significant (p<0.001). Prevalence of ARI was highest in children of illiterate (24.4%) and primary school educated (23.9%) mothers. This difference was not statistically significant.

The above study concluded that strengthening reproductive and child health phase II (RCH-2) or integrated management of childhood illness (IMCI) programs and raising female literacy levels will go a long way in preventing ARI and morbidity amongst children in general. Reorientation of IMCI strategy and training of health workers in peripheral areas regarding identification, management and timely referral of cases of ARI and strong supervision, monitoring and evaluation of rural care health services specifically for ARI are transcendental to improving the quality of the health care centers and decreasing child morbidity and mortality ^17^. A study conducted in eight countries in Latin American (Bolivia, Colombia, Ecuador, Guatemala, Nicaragua, Panama, Peru and Dominican Republic) in 2006 assessed the possible independent and combined action of the parent’s educational level and the economic situation of the family with the risk of diarrheal and respiratory diseases among children under 5 years. For ARIs, the risk of illness decreased as the economic situation of the family improved. However, the mother’s educational level played a contradictory role: the children of mothers with some education had a higher risk of respiratory illnesses than children of mothers without education. This may be because the women with lower educational levels have breastfeed their children for longer or because women with some education could recognize milder respiratory conditions better.

We did not find synergistic effects between the father’s educational level and the family's financial situation.

The Latin American study meantioned above found that better family economic situations reduced the risk of disease in the children of mothers with higher education than in mothers without formal education. Similarly, better education of mothers had a greater protective effect in the children of mothers with a better educational level than in poor families. A better economic situation can allow women to make better choices in regards to child health and helps them to provide their children with better hygiene, a healthy lifestyle and greater access to health care, while educated women living in more severe economic conditions do not always have access to these options for their child’s health. The father’s higher level of education had a protective effect, independent of the economic situation of the family. These results demonstrate that efforts to decrease poverty will have a greater protective effect on child health if they include efforts to improve the educational level of women and girls than if any of these efforts were to be made separately ^18^.

It is believed that the age of the mother may be associated with the level of knowledge about URIs and the use of antibiotics. An analysis of a bivariate study in Spain showed that the level of knowledge was directly related to the age of the person who answered the survey: the older the mother, the higher the level of knowledge (34.5 years compared to 32.8 years) p = 0.003 ^19^.

The results of our study show that the knowledge about resistance and risks of antibiotic use is also not very high for adult mothers, where less than a third had heard of either. This points towards a generally low level of knowledge in the population and the lack of education programs at the community and hospital level.

As described in the results, the physician was stated as the most common source of information about antibiotic resistance by both the adult and adolescent mothers. This shows the potential of physicians as educators, and it should be taken into account when developing interventions targeting the lay population. We believe that the leadership of the medical professional is a key point for the knowledge of the appropriate use of antibiotics.

In a study on the general public’s perceptions and use of antimicrobials in Trinidad and Tobago, it was shown that it was not uncommon for the study participants to incorrectly classify different painkillers and cough and cold formulations as antibiotics ^20^. It is possible that this occurred to a certain extent in this study as well, as the mothers’ understanding of the term "antibiotics" was not investigated. Also, it is possible that some of the participants might have been giving desired answers, rather than truthful ones, when asked about self-medication and early termination of treatment.

Different studies show the importance of knowledge on the appropriate prescribing of antibiotics in children under 5 years, on the economic impact of respiratory infections on the family budget, and on the beliefs of mothers in regards to using antibiotics for acute respiratory infections without a prescription; however, the one factor that contributed to treatment failure was maternal education (p = 0.05) ^21– 23^. This highlights the importance of ensuring a higher level of education in this population group.

The mis- and overuse of antibiotics in the community are behaviors of a complex nature. They are the result of different socioeconomic, cultural, demographic and other factors interacting in ways specific to the local context. In order to identify these factors, multivariate analysis is needed, but even with the significant factors identified, only half the battle is won. In order to be able to develop successful interventions, it is crucial to first understand the mechanisms behind how these factors influence each other and the behaviors and attitudes towards antibiotics.

Little research has been done on the determinants of antibiotic use in the community, and the results are often contradictory ^6, 15, 16, 19– 21^. More attention urgently needs to be given to this part of the combat against antibiotic resistance, if the battle shall be won.

This is a descriptive study, the first addressing this subject in Ecuador as far as the authors are aware. The results show interesting differences in the knowledge and use of antibiotics between teenage and adult mothers. However, there were limitations to what could be statistically analyzed. Therefore a new study design is needed to confirm and quantify the association we have found. Given the current importance of the subject matter and the high prevalence of teenage mothers in our country, this line of research must be continued and requires validation by a more analytical research instrument and the integration of an expert in statistics into the team for the duration of the study.

## References

[1] World Health Organization: The World Health Report 1996--fighting disease, fostering development. *World Health Forum.* 1997;18(1):1–8. 9233055

[2] French GL: The continuing crisis in antibiotic resistance. *Int J Antimicrob Agents.* 2010;36(Suppl 3):S3–S7. 10.1016/S0924-8579(10)70003-0 21129629

[3] Furuya EY Lowy FD: Antimicrobial-resistant bacteria in the community setting. *Nat Rev Microbiol.* 2006;4(1):36–45. 10.1038/nrmicro1325 16357859

[4] Wolff MJ: Use and misuse of antibiotics in Latin America. *Clin Infect Dis.* 1993;17(Suppl 2):S346–S351. 10.1093/clinids/17.Supplement_2.S346 8274599

[5] Multicenter study on self-medication and self-prescription in six Latin American countries. Drug Utilization Research Group, Latin America. *Clin Pharmacol Ther.* 1997;61(4):488–93. 10.1016/S0009-9236(97)90199-5 9129566

[6] Calva J Bojalil R: Antibiotic use in a periurban community in Mexico: A household and drugstore survey. *Soc Sci Med.* 1996;42(8):1121–8. 8737429 10.1016/0277-9536(95)00385-1

[7] Tan YS Hong CY Chong PN: Knowledge that upper respiratory tract infection resolves on its own is associated with more appropriate health-seeking behaviour and antibiotic cognition. *Singapore Med J.* 2006;47(6):518–24. 16752021

[8] Chan GC Tang SF: Parental knowledge, attitudes and antibiotic use for acute upper respiratory tract infection in children attending a primary healthcare clinic in Malaysia. *Singapore Med J.* 2006;47(4):266–70. 16572235

[9] Lee GM Friedman JF Ross-Degnan D: Misconceptions about colds and predictors of health service utilization. *Pediatrics.* 2003;111(2):231–6. 10.1542/peds.111.2.231 12563044

[10] Hart CA Kariuki S: Antimicrobial resistance in developing countries. *BMJ.* 1998;317(7159):647–50. 10.1136/bmj.317.7159.647 9727995 PMC1113834

[11] Clavenna A Bonati M: Drug prescriptions to outpatient children: a review of the literature. *Eur J Clin Pharmacol.* 2009;65(8):749–55. 10.1007/s00228-009-0679-7 19529926

[12] Majeed A Moser K: Age- and sex-specific antibiotic prescribing patterns in general practice in England and Wales in 1996. *Br J Gen Pract.* 1999;49(446):735–6. 10756619 PMC1313505

[13] CEPAR: ENDEMAIN, National Demographic and Maternal and Child Health [National Demographic and Maternal Health Survey, 2004]. Quito: Center for Population Studies and Social Development2004.

[14] OPS/OMS Ecuador's representation. Health situation in Ecuador2006.

[15] Valdez R Ana I Martínez CH: [Educational level of mothers and their knowledge, attitude and practices concerning acute respiratory infections of their children]. *Rev Panam Salud Publica.* 1999;6(6),pp. 400–407[Article in Spanish]. 10659671 10.1590/s1020-49891999001100005

[16] Buñuel ÁJC Fortea GE Cortés MRB: [Antibiotic use in primary care. Do we know what parents think?] *An Pediatr (Barc).* 2004;61(4):298–304. 15456584 10.1016/s1695-4033(04)78392-7

[17] Bipin P Nitiben T Sonaliya KN: A study on prevalence of acute respiratory tract infections(ari) in under five children in urban and rural communities of Ahmedabad district, Gujarat, India. *National Journal of Community Medicine.* 2011;2(2):255 Reference Source

[18] Regional de la Organización Mundial de la Salud para las Américas: Efecto sinérgico del nivel educacional de los padres y la situación económica de la familia en la salud infantil en América Latina. *Rev Panam Salud Publica.* 2006;19(2):pp. 124–125 Reference Source

[19] Parimi N Pinto PLM Prabhakar P: The general public's perceptions and use of antimicrobials in Trinidad and Tobago. *Rev Panam Salud Publica.* 2002;12(1):11–8. 12202020 10.1590/s1020-49892002000700003

[20] Delpiano ML Kabalan BP Diaz VC: [Acute respiratory infections in children of day care center: characteristics and costs]. *Rev Chilena Infectol.* 2006;23(2),pp. 128–133[Article in Spanish]. 10.4067/S0716-10182006000200005 16721446

[21] Gutierrez L: Beliefs of mothers of children between 2 and 5 years on the treatment of acute respiratory infections in San Antonio-Ate Health center. *Lima-Perú.* 2009;pp. 10–120.

[22] Santos M: Compliance the antibiotic therapy in children with pneumonia. *Federal University of Rio de Janeiro.* 1999;pp.145–163 Reference Source

[23] López Y: Incidence of acute respiratory infections in children under five years old. *Electronic Journal of PortalesMedicos.com* 2010 Reference Source

