# Supplementary material for: Differences in antibiotic use and knowledge between adolescent and adult mothers in Ecuador
Source: F1000Res. 2013 Jul 8;2:108. Originally published 2013 Apr 10. [Version 2] doi: 10.12688/f1000research.2-108.v2 (PMC3829124; doi:10.12688/f1000research.2-108.v2)
Supplement: Study questionnaire (Spanish version) — Questionnaire investigating perceptions of disease severity, treatment and antibiotic use by adolescent and adult mothers of children under the age of five. [file f1000research-2-1732-s0001.tgz › CUESTIONARIO_20_FEBRERO_2011.pdf]

FACULTAD DE CIENCIAS MÉDICAS

ReAct ACCION CONTRA LA RESISTENCIA BACTERIANA latinoamérica

PERCEPCIONES DE LAS MADRES Y/O CUIDADORES DE NIÑOS MENORES DE 5 ANOS CON RESPECTO A LAS INFECCIONES REPIRATORIAS EN RELACIÓN CON LA INCLUSIÓN SOCIAL

NOMBRE DEL ENCUESTADOR: ……………………………………………………………………………….....

NOMBRE DE LA UNIDAD OPERATIVA:

- ☐ Hospital Básico de Naranjal
- ☐ Hospital Básico de San Sebastián
- ☐ CS de El Paraíso
- ☐ CS Nabón

RELACION DEL ENCUESTADO CON EL NINO: ……………………………………………………………………

Si la relación del encuestado con el niño es: la Madre o el Principal Responsable del cuidado, se continuará con la encuesta, caso contrario se dará por finalizada la misma.

La categoría étnica no será encuestada a las madres o principales responsables, esta será anotada por simple observación del encuestador

1. ¿A qué categoría étnica pertenece el principal responsable del cuidado del niño?

- ☐ Afroamericano
- ☐ Mestizo
- ☐ Blanco
- ☐ Indígena
- ☐ Montubio

FECHA DE LA ENCUESTA: [ ][ ] Día [ ][ ] Mes [ ] Año

NUMERO DE ENCUESTA: [ ][ ][ ][ ]

Por favor NO llenar el número de encuesta pues esta será empleado durante la tabulación.

INSTRUCCIONES GENERALES

La mayoría de preguntas son de opción múltiple, en donde una opción o más podrán ser señaladas. Por favor use una "X" o un "visto bueno" para indicar las respuestas, así: ☒ ☑

Si comete un error, por favor rellene el cuadro de esta forma: ■, entonces marque la opción correcta.

En algunas preguntas existe una línea, en la cual Usted deberá escribir con letra imprenta y legible la respuesta, y la clasificación será realizada por los responsables de la investigación en cada unidad de Salud.

CUESTIONARIO

A. Características básicas del Niño o Niña:

1. Fecha de nacimiento del niño:

En la primera parte se anotará la fecha de nacimiento del niño con exactitud, en la siguiente sección se detallará la edad en años y meses y finalmente se clasificará.

Fecha de Nacimiento: ☐☐ Día ☐☐ Mes ☐ Año

Edad en años y meses: ☐ Años ☐☐ Meses

2. Clasificación edad:

☐ < 2 meses

☐ 2 meses – 11 meses

☐ 12 meses – 5 años

3. ¿Sexo del niño?

☐ Masculino ☐ Femenino

B. Características del Principal responsable del cuidado del niño:

1. ¿Quién es el principal responsable del cuidado diario del niño?

Se tomará como principal responsable a la persona quien se encargue de llevar al niño a la guardería, acompañamiento al niño durante problemas médicos, esparcimiento del niño durante los fines de semana (sacarlo a jugar, llevarle al parque)

- ☐ Madre
- ☐ Padre
- ☐ Familiar
- ☐ Otra persona

2. Si no es la madre, ¿Cuál es la razón?

- ☐ Ella vive y/o trabaja en otro lugar dentro o fuera del país, pero aporta económicamente para la crianza del niño.
- ☐ No está viva.
- ☐ Abandonó el hogar y al niño, por lo tanto **no** aporta económicamente a la crianza del mismo.
- ☐ Otra razón.

3. Edad del principal responsable del cuidado del niño: ☐☐

4. Estado civil del principal responsable del cuidado del niño:

- ☐ Casada (o)
- ☐ Soltera (o)
- ☐ Unida (o)
- ☐ Viuda (o)
- ☐ Separada (o)
- ☐ Divorciada (o)
- ☐ No responde

5. Años de escolaridad del principal responsable del cuidado del niño:

- ☐ Ninguno
- ☐ Escuela completa
- ☐ Escuela incompleta
- ☐ Colegio completo
- ☐ Colegio incompleto

- ☐ Universidad completa
- ☐ Universidad incompleta
- ☐ Posgrados, maestrías
- ☐ Otro

6. Ocupación del principal responsable del cuidado del niño:

- ☐ **Obrero** *(Trabajador con contrato permanente por más de 3 meses en empresas donde haya >10 trabajadores incluye a trabajadores de empresas estatales siempre y cuando no cumpla labores administrativas, también incluye obreros que trabajan en casa pero para empresas ej: sastres, zapateros).*
- ☐ **Artesano y/o pequeño industrial** *(Maestro independiente dueño de una unidad productiva con 2 o 10 trabajadores, ej: carniceros, modistas, panaderos, peluqueros, técnicos de radio y tv)*
- ☐ **Semiasalariado** *(Trabajador irregular con labores inestables y de comercio menor, ej: cuidadores de carros, vendedor ambulante, cocinera, lavanderas, aprendiz de artesanía, cargadores, peones de construcción).*
- ☐ **Empleado Público** *(Empleado de empresa estatal no directamente vinculado con fábricas e industrias, ej: secretaria, oficinista, profesor, guardián, chofer, jardinero).*
- ☐ **Empleado empresa Privada** *(Personal técnico – administrativo de empresas privadas, con >10 empleados con contratos permanentes, aquí se INCLUYE a empleados técnicos – administrativos de empresas estatales productivas como Fábricas, minas)*
- ☐ **Comerciante menor** *(Personas que basan su economía en torno a la compra de productos elaborados o semielaborados y su posterior venta en local estable con el apoyo de su familia y/o no más de 5 vendedores, ej: tendero, pequeña botica, ferretería, dueño bazar).*
- ☐ **Administra propia empresa** *(Persona dueña de una fábrica, mina, empresa de seguros, empresa agropecuaria, con >10 trabajadores contratados.*
- ☐ **Profesional independiente** *(Individuo con alta calificación universitaria o técnica que ejerce libremente su profesión, ej: medico, abogado, odontólogo, obstetras, topógrafo)*
- ☐ **Pequeño trabajador agrícola** *(Es el trabajador, pero no obrero ni jornalero, es decir no trabaja ni en empresa agroindustrial ni en relación transitoria por jornal y cuya subsistencia se basa en el trabajo como propietario de una parcela, mimbro de alguna cooperativa, sembrador al partir).*
- ☐ **Desocupado** *(habiendo sido trabajador activo ha perdido el trabajo y está sin laborar desde al menos 1 semana)*
- ☐ **Otros**

C. Características del Segundo responsable del cuidado diario del niño

1. ¿Quién es el segundo responsable del cuidado diario del niño?

- ☐ Madre
- ☐ Padre
- ☐ Familiar
- ☐ Otra persona
- ☐ No existe una segunda persona responsable del cuidado del niño **(pasar sección D)**

2. Si no es el padre, ¿Cuál es la razón?

- ☐ Él vive y/o trabaja en otro lugar dentro o fuera del país, pero aporta económicamente para la crianza del niño.
- ☐ No está vivo.
- ☐ Abandonó el hogar y al niño, por lo tanto **no** aporta económicamente a la crianza del mismo.
- ☐ Otra razón.

3. Edad del segundo responsable del cuidado diario del niño: ☐☐

4. ¿A qué categoría étnica pertenece el segundo responsable del cuidado diario del niño?

- ☐ Afroamericano
- ☐ Mestizo
- ☐ Blanco
- ☐ Indígena
- ☐ Montubio

5. Estado civil del segundo responsable del cuidado diario del niño:

- ☐ Casado
- ☐ Soltero
- ☐ Unido
- ☐ Viudo
- ☐ Separado
- ☐ Divorciado
- ☐ No responde

6. Años de escolaridad del segundo responsable del cuidado diario del niño:

- ☐ Ninguno
- ☐ Escuela completa
- ☐ Escuela incompleta
- ☐ Colegio completo
- ☐ Colegio incompleto
- ☐ Universidad completa
- ☐ Universidad incompleta
- ☐ Posgrados, maestrías
- ☐ Otro

7. Ocupación del segundo responsable del cuidado diario del niño:

- ☐ **Obrero** *(Trabajador con contrato permanente por más de 3 meses en empresas donde haya >10 trabajadores incluye a trabajadores de empresas estatales siempre y cuando no cumpla labores administrativas, también incluye obreros que trabajan en casa pero para empresas ej: sastres, zapateros).*
- ☐ **Artesano y/o pequeño industrial** *(Maestro independiente dueño de una unidad productiva con 2 o 10 trabajadores, ej: carniceros, modistas, panaderos, peluqueros, técnicos de radio y tv)*
- ☐ **Semiasalariado** *(Trabajador irregular con labores inestables y de comercio menor, ej: cuidadores de carros, vendedor ambulante, cocinera, lavanderas, aprendiz de artesanía, cargadores, peones de construcción).*
- ☐ **Empleado Público** *(Empleado de empresa estatal no directamente vinculado con fábricas e industrias, ej: secretaria, oficinista, profesor, guardián, chofer, jardinero).*
- ☐ **Empleado empresa Privada** *(Personal técnico – administrativo de empresas privadas, con >10 empleados con contratos permanentes, aquí se INCLUYE a empleados técnicos – administrativos de empresas estatales productivas como Fábricas, minas)*
- ☐ **Comerciante menor** *(Personas que basan su economía en torno a la compra de productos elaborados o semielaborados y su posterior venta en local estable con el apoyo de su familia y/o no más de 5 vendedores, ej: tendero, pequeña botica, ferretería, dueño bazar).*
- ☐ **Administra propia empresa** *(Persona dueña de una fábrica, mina, empresa de seguros, empresa agropecuaria, con >10 trabajadores contratados.*
- ☐ **Profesional independiente** *(Individuo con alta calificación universitaria o técnica que ejerce libremente su profesión, ej: medico, abogado, odontólogo, obstetras, topógrafo)*
- ☐ **Pequeño trabajador agrícola** *(Es el trabajador, pero no obrero ni jornalero, es decir no trabaja ni en empresa agroindustrial ni en relación transitoria por jornal y cuya subsistencia se basa en el trabajo como propietario de una parcela, mimbro de alguna cooperativa, sembrador al partir).*
- ☐ **Desocupado** *(habiendo sido trabajador activo ha perdido el trabajo y está sin laborar desde al menos 1 semana)*
- ☐ **Otros**

D. Inserción social del núcleo familiar

1. Número total de niños (as) <5 años en la familia:

☐☐

2. Cuántos viven en la casa que compartan la misma olla: ☐☐

3. ¿Dónde vive la familia?:

Para esta clasificación cada responsable entregara a los encuestadores una lista de las zonas urbanas y rurales

- ☐ Urbano
- ☐ Rural

4. ¿Cuántos dormitorios tiene la casa/departamento?

☐☐

5. ¿Qué servicios básicos posee en su vivienda?

Se puede marcar más de una opción

- ☐ Electricidad
- ☐ Agua potable
- ☐ Teléfono
- ☐ Alcantarillado
- ☐ Tv cable
- ☐ Internet
- ☐ Todas

6. ¿Quién aporta mayor dinero para la economía de la familia?

- ☐ El padre
- ☐ La madre
- ☐ Otra persona

7. ¿Hay alguna otra fuente de ingreso económico para la familia aparte de los anteriormente nombrados?

☐ Si ☐ No

8. En caso afirmativo, designe cual:

- ☐ Familia (sea o no del extranjero)
- ☐ Estado (Bono, curia, etc)

9. Principal fuente de ingreso de la familia:

Por "soporte de Familia de los Padres" se entiende cuando los padres de la Madre, Padre o cuidador generan ingresos al hogar del niño. En el caso de "soporte de familia de otra persona" es lo mismo pero referido a cualquier persona aparte de padres o cuidadores, ej: tíos, sobrinos, vecinos, madrinas, padrinos

- ☐ Sueldo o salario
- ☐ Alquiler de propiedad
- ☐ Negocio particular
- ☐ Soporte de la familia de los padres
- ☐ Soporte de la familia de otra persona

10. ¿Qué cantidad aproximada de dólares tiene como ingreso mensualmente la familia, contando bono, ayuda familiar e ingreso propio del hogar?

- ☐ <263
- ☐ 264 – 463
- ☐ 464 – 663
- ☐ 664 – 863
- ☐ > 864

E. Conocimiento de la IRA

En esta sección es importante que la madre **NO** sepa las opciones que tiene, por el contrario, que las mismas partan de su conocimiento y actitud verdadera, esta respuesta se escribirá de forma clara y legible y abajo se clasificara dentro de la (s) opción (es) más adecuada (s).

1. ¿Porque cree que su niño se enferma de las Vías respiratorias?

……...

……...

- ☐ El clima
- ☐ La comida
- ☐ Los gérmenes
- ☐ Falta de aseo en lugares de cuidado
- ☐ No sé
- ☐ Otro

2. ¿Cómo cree Ud. que las enfermedades respiratoria en los niños deben ser tratadas para mejorarse más rápido?

………

……...

- ☐ Tratamiento natural
- ☐ Antibióticos
- ☐ Medicamentos para la fiebre
- ☐ Medicamentos para la tos y/o flema
- ☐ Ungüentos corporales
- ☐ Visita medica
- ☐ Otro

3. ¿Cuándo cree que la enfermedad respiratoria de su niño es grave y necesita atención de un médico?

………

………

- ☐ Tiene fiebre muy alta
- ☐ Vomito persistente
- ☐ Dificultad para respirar
- ☐ No se alimenta
- ☐ Ruidos extraños al respirar (ronquido pecho)
- ☐ Cambios coloración de la piel
- ☐ Otro

4. ¿Cuándo la enfermedad respiratoria no es grave y no necesita ir al médico, que tratamiento da normalmente?

………

………

- ☐ Tratamiento natural
- ☐ Antibióticos
- ☐ Medicamentos para la fiebre
- ☐ Medicamentos para la tos y/o flema
- ☐ Ungüentos corporales
- ☐ Otro

F. Comportamiento de la IRA

1. ¿Su niño ya ha tenido alguna enfermedad respiratoria antes?

- ☐ Sí (pasar a la siguiente pregunta)
- ☐ No (Pasar a la pregunta número 3)

2. ¿Su niño ha tenido una enfermedad respiratoria en los 3 últimos meses?

☐ Si ☐ No

3. ¿Que hizo la última vez que su niño presentó enfermedad respiratoria?

En esta pregunta es importante que la madre **NO** sepa las opciones que tiene, por el contrario, que las mismas partan de su conocimiento y actitud verdadera, esta respuesta se escribirá de forma clara y legible y abajo se clasificará dentro de la (s) opción (es) más adecuada (s).

……

……

- ☐ Visitó al Médico y/o Unidad de Salud
- ☐ Farmacia
- ☐ Familia
- ☐ Tienda
- ☐ Otra cosa
- ☐ Nada (Pasar a la pregunta número 8)

4. Describa el tratamiento que empleó la última vez que su niño presentó enfermedad respiratoria.

En esta pregunta se recomienda escribir el nombre del (los) medicamentos que la madre enumere, para luego clasificarlos en los grupos de la zona inferior.

……..........

……..........

- ☐ Tratamiento antibiótico
- ☐ Tratamiento antipirético y/o analgésico
- ☐ Tratamiento mucolítico y/ o antitusígeno
- ☐ Tratamiento antihistamínico
- ☐ Ungüentos corporales
- ☐ Otro

5. ¿Si fue a la farmacia o dió medicamentos en casa sin ir al médico, cuáles razones tuvo para hacerlo?

En esta pregunta es importante que la madre **NO** sepa las opciones que tiene, por el contrario, que las mismas partan de su conocimiento y actitud verdadera, esta respuesta se escribirá de forma clara y legible y abajo se clasificará dentro de la (s) opción (es) más adecuada (s).

En caso de haber asistido al Médico y/o **no** haber dado ningún medicamento en casa, esta pregunta quedará en blanco

………..........

………..........

- ☐ No tuve tiempo para ir al medico
- ☐ No hubo acceso a la atención médica por falta de turnos disponibles
- ☐ No tuve dinero para ir al médico
- ☐ La condición **no** estuvo tan grave
- ☐ Tenía medicamentos en casa
- ☐ Terceras personas me aconsejaron algún tipo de remedio
- ☐ Otro

6. ¿Qué medida cree que es mejor para prevenir la enfermedad respiratoria?

En esta pregunta es importante que la madre **NO** sepa las opciones que tiene, por el contrario, que las mismas partan de su conocimiento y actitud verdadera, esta respuesta se escribirá de forma clara y legible y abajo se clasificará dentro de la (s) opción (es) más adecuada (s).

………..........

………..........

- ☐ Lavado de manos
- ☐ Uso de pañuelos
- ☐ Evitar llevar al niño a la escuela o guardería si está enfermo
- ☐ No dar ningún medicamento
- ☐ Acudir a visita médica mensual
- ☐ Buena alimentación
- ☐ Otro

7. ¿Esta vez, cuantos días esperó antes de acudir a esta institución? ☐☐

8. ¿Esta vez, ha dado alguno medicamento a su niño antes de la visita?

☐ Si ☐ No

9. En caso afirmativo, indique ¿Cuál?

En esta pregunta se recomienda escribir el nombre del (los) medicamentos que la madre enumere, para luego clasificarlos en los grupos de la zona inferior.

………......

………......

- ☐ Antibióticos
- ☐ Medicamentos antipiréticos y/o analgésicos
- ☐ Medicamentos Mucolíticos y/ o antitusígenos
- ☐ Medicamentos antihistamínicos
- ☐ Ungüentos corporales
- ☐ Remedio natural
- ☐ Otro

G. Comportamiento de los Antibióticos

1. ¿Alguna vez ha obtenido/comprado antibióticos sin ir al médico?

- ☐ Si (Pasar a la siguiente pregunta)
- ☐ No (Pasar a la pregunta número 3)

2. En caso afirmativo, indique: ¿a quién o en dónde adquirió los antibióticos la última ocasión?

- ☐ En la Farmacia
- ☐ En la tienda
- ☐ De un amigo/vecino
- ☐ Otro

3. ¿Usted conoce algunos de los efectos que produce el uso de antibióticos?

☐ Si ☐ No (Pasar a la pregunta número 5)

4. En caso afirmativo, ¿Cuál?

En esta pregunta es importante que la madre **NO** sepa las opciones que tiene, por el contrario, que las mismas partan de su conocimiento y actitud verdadera, esta respuesta se escribirá de forma clara y legible y abajo se clasificará dentro de la (s) opción (es) más adecuada (s).

………......

………......

- ☐ Resistencia Bacteriana
- ☐ Alergias
- ☐ Efectos Adversos (efectos secundarios)
- ☐ Tolerancia a los medicamentos
- ☐ Otros
- ☐ No sabe

5. ¿Cuándo el médico prescribe antibióticos a su niño, termina el tratamiento o lo suspende antes? Es decir cumple con todos los días recomendados por el médico para el tratamiento?

- ☐ Sí (Pasar a la pregunta número 7)
- ☐ No (Pasar a la siguiente pregunta)

6. En caso negativo. ¿Cuál fue la razón?

En esta pregunta es importante que la madre **NO** sepa las opciones que tiene, por el contrario, que las mismas partan de su conocimiento y actitud verdadera, esta respuesta se escribirá de forma clara y legible y abajo se clasificará dentro de la (s) opción (es) más adecuada (s).

………......

………......

- ☐ Prefería guardar los antibióticos para otra vez
- ☐ Compartí los antibióticos con otro niño
- ☐ No tenía dinero para terminar el tratamiento durante todos los días
- ☐ Mi niño se sentía mejor
- ☐ No le gusta a mi niño los medicamento
- ☐ El medicamento le da efectos colaterales
- ☐ Olvido
- ☐ Otro

7. ¿Cuándo cree Usted que está indicado usar antibióticos?

En esta pregunta es importante que la madre **NO** sepa las opciones que tiene, por el contrario, que las mismas partan de su conocimiento y actitud verdadera, esta respuesta se escribirá de forma clara y legible y abajo se clasificará dentro de la (s) opción (es) más adecuada (s).

……......

……......

- ☐ Fiebre
- ☐ Dolor de cabeza
- ☐ Dolor de oídos
- ☐ Gripe, resfrío e infecciones virales
- ☐ Congestión Nasal
- ☐ Dolor de garganta
- ☐ Infecciones bacteriana
- ☐ Cuando el Medico lo ordena
- ☐ Otro

8. ¿Alguna vez ha oído hablar de bacterianas resistentes de los antibióticos?

- ☐ Si (Pasar a la siguiente pregunta)
- ☐ No (Se dará por finalizada la encuesta)

9. En caso afirmativo. ¿Dónde?

- ☐ Del médico
- ☐ De un amigo y/o vecino
- ☐ En el periódico
- ☐ En la televisión
- ☐ En la radio
- ☐ Otro

10. ¿Qué cree que deberíamos hacer para evitar la Resistencia Bacteriana?

En esta pregunta es importante que la madre **NO** sepa las opciones que tiene, por el contrario, que las mismas partan de su conocimiento y actitud verdadera, esta respuesta se escribirá de forma clara y legible y abajo se clasificará dentro de la (s) opción (es) más adecuada (s).

………......

………......

- ☐ No usar antibióticos **sin** acudir a un medico
- ☐ No automedicarse
- ☐ No dar antibióticos a los animales para engordarlos
- ☐ No sabe
- ☐ Otro
