# Supplementary material for: Differences in antibiotic use and knowledge between adolescent and adult mothers in Ecuador
Source: F1000Res. 2013 Jul 8;2:108. Originally published 2013 Apr 10. [Version 2] doi: 10.12688/f1000research.2-108.v2 (PMC3829124; doi:10.12688/f1000research.2-108.v2)
Supplement: Peer review history

# Peer review: Referee response for version 2

**DOI:** 10.5256/f1000research.1732.r3977

**Published:** 2014-03-05

**Authors:** Liliana Clara

**Affiliations:** 1 Hospital Italiano de Buenos Aires, Buenos Aires, Argentina

I have read this submission. I believe that I have an appropriate level of expertise to confirm that it is of an acceptable scientific standard.

# Peer review: Referee response for version 2

**DOI:** 10.5256/f1000research.1732.r1108

**Published:** 2013-07-17

**Authors:** Andrew Gilbert

**Affiliations:** 1 Division of Health Sciences, School of Pharmacy and Medical Sciences, University of South Australia, Adelaide, SA, Australia

I am happy with the changes made to the paper and the clarification that this was a descriptive study. I congratulate the authors for the effort but into this study and hope that a follow-up study will be undertaken to further explore this important area in public health.

I have read this submission. I believe that I have an appropriate level of expertise to confirm that it is of an acceptable scientific standard.

# Peer review: Referee response for version 1

**DOI:** 10.5256/f1000research.1208.r994

**Published:** 2013-06-10

**Authors:** Liliana Clara

**Affiliations:** 1 Hospital Italiano de Buenos Aires, Buenos Aires, Argentina

This article shows that education efforts should be targeted to concentrate more on these specific age groups.

I have read this submission. I believe that I have an appropriate level of expertise to confirm that it is of an acceptable scientific standard.

# Peer review: Referee response for version 1

**DOI:** 10.5256/f1000research.1208.r891

**Published:** 2013-04-15

**Authors:** Andrew Gilbert

**Affiliations:** 1 Division of Health Sciences, School of Pharmacy and Medical Sciences, University of South Australia, Adelaide, SA, Australia

This is an interesting and important topic area to study. The paper is well written and the conclusions match the results of the data analysis. Basically it is stating the obvious: Older, more experienced mothers, who are generally better educated, have more money and have more children are better at using antibiotics and know about antibiotic resistance. The paper would benefit from having a statistician provide advice on data analysis; a better story could be told about the influence of age etc if logistic regression models were used.

One of main concerns in the interpretation provided in that it has missed the obvious question about, “were the older mothers the same as the young mums in terms of actions, knowledge and use of antibiotics. How would the older mums compare if those with only one child were compared with the younger mums; ie is knowledge about antibiotics and obtaining antibiotics for medical practitioners learnt “on the job”. Planned regression modelling can answer questions such as this.

If no statistical support is available, the authors should at least acknowledge this and include in the discussion a statement, such in the paragraph above, on weaknesses of this study. Any future study should include a statistician at the very first study planning session to ensure adequate data are collected via the questionnaire to answer the research question(s), and the appropriate statistical procedures. There is no mention in the study of basic statistical requirements such as a power calculation, for example.

I have read this submission. I believe that I have an appropriate level of expertise to confirm that it is of an acceptable scientific standard, however I have significant reservations, as outlined above.
